# Stimulus Familiarity Affects Perceptual Restoration in the European Starling (*Sturnus vulgaris*)

**DOI:** 10.1371/journal.pone.0005974

**Published:** 2009-06-24

**Authors:** Folkert Seeba, Georg M. Klump

**Affiliations:** Animal Physiology and Behavior Group, Institute of Biology and Environmental Sciences, Carl von Ossietzky University Oldenburg, Oldenburg, Germany; University of Lethbridge, Canada

## Abstract

**Background:**

Humans can easily restore a speech signal that is temporally masked by an interfering sound (e.g., a cough masking parts of a word in a conversation), and listeners have the illusion that the speech continues through the interfering sound. This perceptual restoration for human speech is affected by prior experience. Here we provide evidence for perceptual restoration in complex vocalizations of a songbird that are acquired by vocal learning in a similar way as humans learn their language.

**Methodology/Principal Findings:**

European starlings were trained in a same/different paradigm to report salient differences between successive sounds. The birds' response latency for discriminating between a stimulus pair is an indicator for the salience of the difference, and these latencies can be used to evaluate perceptual distances using multi-dimensional scaling. For familiar motifs the birds showed a large perceptual distance if discriminating between song motifs that were muted for brief time periods and complete motifs. If the muted periods were filled with noise, the perceptual distance was reduced. For unfamiliar motifs no such difference was observed.

**Conclusions/Significance:**

The results suggest that starlings are able to perceptually restore partly masked sounds and, similarly to humans, rely on prior experience. They may be a suitable model to study the mechanism underlying experience-dependent perceptual restoration.

## Introduction

While listening to your friend at a cocktail party telling a story, your perception is only mildly disturbed if some other person next to you coughs. Although the cough is a sound that completely masks the speech to which you are listening, you may even have the percept of hearing a continuous word rather than one that is interrupted by the disturbing sound. Warren [1] observed that missing parts of a speech signal can be restored in perception if the temporal gap in the speech sound is filled by a noise masker. Since there was no speech sound in the noise-filled gap, the auditory percept of a continuing speech is an illusion. Warren [2] used the expression temporal induction, and in the case of speech sounds phonemic restoration and suggested that principles developed within the framework of Gestalt theory that has been widely applied in the context of the segregation of sources in auditory scene analysis can account for the perceptual restoration [3], [4]. Although the effect of temporal induction is so important for everyday communication, the underlying mechanisms are only partly understood.

So far, animal studies investigating the mechanisms underlying temporal induction have mostly used simple artificial stimuli. Temporal induction has been demonstrated in behavioral experiments for pure tones (Macaque monkey [5], European starling [6]) and frequency modulated tones (cat [7]). Also more complex stimuli have been used such as species specific calls in behavioral experiments with monkeys (cotton-top tamarin [8], macaque [5]) and a single vocalization of a starling and a budgerigar in behavioral experiments with starlings [9]. These experiments indicate that temporal induction in animal models is found both for stimuli with simple and with complex features.

Human psychophysical experiments suggest that prior knowledge of the language reflected in the form of lexical activation affects the perceptual restoration for complex speech sounds. In these experiments, Samuel [10] compared the auditory restoration with real words and pseudo words and found a larger amount of restoration in the real words. He also found a better restoration for words that were presented to the subjects a number of times before the test. Both results indicate that previous experience with the stimuli improves the restoration. Furthermore, experiments on speech reception in background noise reported that native listeners (i.e., subjects that listen to speech sounds of their mother tongue) have an advantage over non-native listeners in identifying keywords in sentences masked by background noise [11]. This observation suggests that the learned neural representation improves the recognition of partially masked speech signals.

Here we present data from a behavioral experiment in the European starling (*Sturnus vulgaris*) indicating that this songbird species provides an excellent model to study such an interaction between the learned neural representation of complex signals and their perceptual restoration which is a hallmark of perceptual restoration in humans [10]. Songbirds are the prime model for studying the processes underlying vocal learning in which a learned neural representation of the signals guides the vocal development [12], [13]. The processes in the acquisition of birdsong signals show many parallels to the processes in the acquisition of speech in humans [14]. Starlings have a highly complex song with a hierarchical structure that is similar to the structure found in human language [15], [16]. The sequence of motifs and notes (subunits of motifs) in the starling song is determined by learned rules that are comparable to the grammar of human language [17]. Learning modifies the neural representation of the perceived song and its elements in the starling forebrain creating templates for the analysis of song signals [18], [19]. These templates that can be represented by neurons acting as detectors for complex features of a sound could form the neural substrate for perceptual restoration in a similar way as the preexisting lexical representation affects restoration in humans [10]. Therefore, we predict that familiarity with a song signal will affect perceptual restoration of motifs in the starling as does the familiarity with phonemes and words affect perceptual restoration in humans [10], [20]).

**Figure 1:**
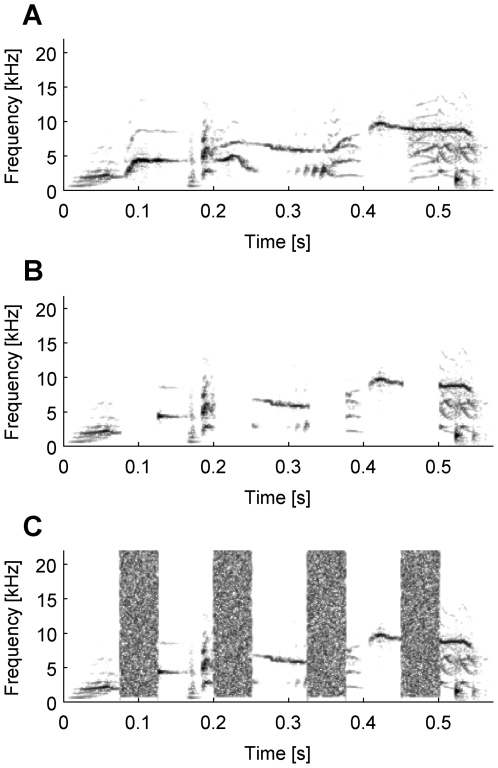
Spectrograms of an exemplary stimulus set. Spectrograms (spectral power indicated by darkness of shading plotted as a function of time vs. frequency) of an exemplary complete motif and its modifications; (A) complete motif, (B) the same motif as in (A) with 50 ms silent gaps introduced every 75 ms, (C) the same as in (B), but now the gaps are filled with band-passed noise to produce a noise motif.

To reveal the effect of familiarity on perceptual restoration we trained starlings in a general same/different-paradigm to report salient changes in the structure of successive sounds. The basic training, in which the birds generalized the task from one stimulus set to the next used exclusively pure tones, sinusoidal amplitude modulated noise or starling whistle motifs that were not used as test stimuli in the subsequent experiment. The birds were, thus, not trained to discriminate the song motifs that were used in the subsequent experiments. These song motifs either were completely new to the starlings (i.e., unfamiliar), or they were songs from cage mates or the bird's own song (i.e., familiar). To test whether starlings have an auditory illusion similar to perceptual restoration of speech signals they had to discriminate three different modifications of one motif in each experiment:

(A) a complete starling song motif (complete motif), (B) the same song motif with silent gaps (gap motif), and (C) the same motif with the gaps filled with noise (noise motif, see Fig. 1.

In the behavioral discrimination experiments, one of the three stimuli was presented as a repeating background and a response to any stimulus deviating from the background (deviator) was rewarded. The response latency was determined that indicates the salience of the difference between the background stimulus and the deviator (i.e., a shorter latency indicates a more salient difference [21]). If perceptual restoration occurs in the noise stimulus but not in the gap stimulus, we would expect a larger response latency for discrimination between the complete and the noise stimulus than for discrimination between the complete and the gap stimulus since the restoration would make the modified noise motif more similar to the complete motif.

## Results

Data from the discrimination of 20 motifs were collected, 10 were familiar and 10 unfamiliar to the birds. Every modification of a motif served both as the background and the deviator, and 30 measurements of response latencies were obtained for every deviator/background combination. The mean response latencies for discriminating the modifications of each motif were combined into a response matrix for every bird. An example for a response matrix pooling data from 4 individual birds obtained in one experiment is shown in Table 1. The birds responded faster when discriminating between gap motifs and complete motifs (1315 ms±200 ms, mean±SE) than when discriminating between noise and complete motifs (1573 ms±203 ms). The example indicates that the difference between gap motifs and complete motifs is more salient than between noise motifs and complete motifs. A similar result is observed if the response latencies for discriminating a gap motif or a noise motif from a background of complete motifs are compared now analyzing data from all 20 experiments performed with the four birds. A general linear mixed model ANOVA was used for evaluating the effects of the fixed factors familiarity (familiar, unfamiliar) and motif modification (gap motif, noise motif) on the response latencies for making the discrimination from a background of complete motifs. The subject identity and the motif number were included in the analysis as random covariates. Both familiarity (p<0.001) and motif modification (p = 0.043) had a significant effect on response latency and there was a significant interaction (p = 0.004) between both effects. Discriminating a familiar modification from a background took much longer (on average 1349 ms) than discriminating an unfamiliar modification from the background (on average 979 ms). Furthermore, birds reported the occurrence of gap motifs in the background of complete motifs faster (average response latency 1110 ms) than the occurrence of noise motifs (average response latency 1217 ms). These average response latencies were shorter than the response latencies scored in control trials in which no change occurred (on average these were 1946 ms and 1988 ms in the familiar and unfamiliar condition, respectively, which is close to the maximum response latency of 2000 ms that was scored if no response occurred). The significant interaction reflects that there was no significant difference between the reporting of the two modified motif types in the unfamiliar condition (p = 0.452, average response latencies for discriminating gap and noise motifs from the from the background of complete motifs were 1002 and 955 ms, respectively), whereas there was a highly significant difference in the response latencies for reporting the two modified motif types in the familiar condition (p = 0.002, average response latencies for discriminating gap and noise motifs from the from the background of complete motifs were 1219 and 1478 ms, respectively). In a more detailed analysis differentiating between levels of familiarity, we found no difference in the response for song elements that the birds had heard from cage mates and song elements that they sang themselves (i.e., the bird's own song). In general, it took the birds longer to discriminate the gap or noise modifications from a complete motif if the motif was familiar than if it was unfamiliar. Average response latencies for discriminating familiar and unfamiliar gap motif modifications from complete motifs were 1219 and 1002 ms, respectively (p = 0.0042). Average response latencies for discriminating familiar and unfamiliar noise motif modifications from complete motifs were 1478 and 955 ms, respectively (p<0.0005).

**Table 1:** Exemplary pooled half matrix.

Background/Deviator	Gap	Noise	Complete
Gap	1891		
	±56		
Noise	1319	1816	
	±202	±161	
Complete	1315	1573	1902
	±200	±203	±77

A pooled half matrix of response latencies to one stimulus set out of one experiment from four individual birds with mean±standard error values in ms for every background/deviator combination.

**Figure 2:**
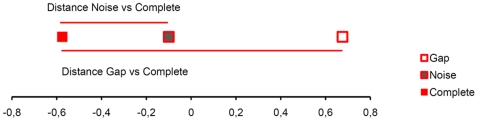
Exemplary multidimensional scaling result. An exemplary multidimensional scaling result derived from the data shown in Tab. 1: the scaling result indicates a smaller distance between the perceived complete and noise stimuli than between the perceived complete and gap stimuli.

To further evaluate the salience of the discrimination, a perceptual map for each of the 20 experiments was constructed using the multidimensional scaling procedure PROXSCSCAL (SPSS, SPSS Inc.). The solution for the exemplary matrix of response latencies presented above is shown in Fig. 2. Since on average already the first dimension accounted for 87.4% and 89.0% of the variance for familiar and unfamiliar motifs, respectively, we obtained only one-dimensional solutions of the Proxscal algorithm and used these data to calculate the perceptual distances. The longer reaction times of the four birds in discriminating between complete and noise stimuli than between complete and gap stimuli resulted in a smaller distance between the representation of complete and noise motifs than for the complete and gap motifs.

**Figure 3:**
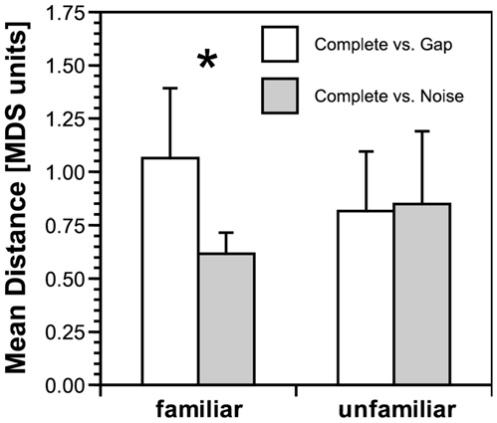
Mean perceptual distance in relation to the stimulus modification and the familiarity of the motif. For familiar motifs the perceptual distances (MDS units = multidimensional scaling units) was significantly larger between the complete and the gap stimulus than between the complete and the noise stimulus (* indicates p<0.005), but there was no such difference between unfamiliar stimuli.

The mean distances for discriminating modifications of 10 familiar and the 10 unfamiliar motifs are plotted in Fig. 3. In the familiar motif condition, the complete and gap motifs were significantly further separated than complete and noise motifs (p = 0.003; t_9_ = 3.95, t-test, two-tailed). In the unfamiliar motif condition no such difference in perception could be shown (p = 0.84; t_9_ = 0.208, t-test, two-tailed).

## Discussion

Applying a behavioral paradigm to determine the salience of a perceptual difference based on response latencies and employing a multidimensional scaling analysis we demonstrate that European starlings perceive a complete motif from a familiar starling song as being more similar to a rendition of the motif in which parts are replaced by broadband noise than to a rendition of the motif in which silent intervals are introduced instead. This difference is expected if the noise leads to a perceptual restoration of the motif by the auditory system. For unfamiliar motifs from a starling song no such difference was observed suggesting that perceptual restoration occurs to a much smaller extent in that case.

The results are consistent with the notion that the birds may have an illusionary percept of the restored sounds that is similar to the auditory illusion found for the perceptual restoration in speech sounds by human subjects [1], [2], [3], [10]. The finding that the birds were significantly faster to discriminate a modification of a familiar motif with gaps from the complete motif than to discriminate a familiar motif with noise-filled gaps from the complete motif indicates that the latter difference is less salient to the birds. This view is supported by the results of the multi-dimensional scaling analysis that also provide evidence for a smaller perceptual distance between the familiar motif with noise-filled gaps and the complete motif than between the familiar motif with silent gaps and the complete motif. The result that the birds in general took longer to discriminate familiar motif modifications than unfamiliar motif modifications may indicate familiarity per se provides for some restoration. The observed effect of familiarity in the starling parallels the effects that prior experience with the stimuli has for the perceptual restoration in human subjects [10].

By shaping the birds' behavioral response in the baseline training with stimuli that were different from the motifs used in the main experiments we can assume that the observed differences in the discrimination of the motif modifications occurred spontaneously and were not due to specific discrimination training. In addition, the similarity in the distances from both the gap and the noise motif to the complete motif that was observed in the case of unfamiliar motifs indicates that the presentation of the interspersed (see method section) noise did not bias the birds' response. Obviously, the repeated presentation of the motifs during the main experiment did not result in a gain in the familiarity of the stimuli that would have affected the outcome of the experiment.

For the familiarity to become effective, it may require processes that occur over a longer time scale or involve contextual conditions such as social interactions with conspecifics that were not provided in the current experiment. The familiar songs were songs that the birds could hear when listening to conspecifics during many months before the experiment started when they were housed together and these motifs also included motifs that they had acquired during song learning. In such conditions, starlings are known to learn new song elements [e.g., 22], [23] from conspecifics.

Our results raise questions about the neural mechanism and the site in the auditory system where such a perceptual restoration of complex song elements may occur. The song elements are much more complex than the tonal signals for which temporal induction has been demonstrated both behaviorally and at the level of neurons in the primary auditory cortex [7], [24]). In contrast to the species-specific vocalizations used in other studies that probably are not acquired by vocal learning [5], [8], the song elements presented to the starlings in the current study are known to be learned [22] and previous experience of the birds with the stimuli and their familiarity may affect their neural processing and thus the mechanisms underlying the perceptual restoration. The starling's auditory forebrain area that corresponds to the primary auditory cortex of mammals is the Field L complex [25]. In this area, an experience dependent change in the neural representation of vocalizations has been demonstrated in starlings and other songbirds allowing the sensory processing to adapt to the statistics of the stimulus features [26], [27]. These representations reflect different stimulus characteristics [28], [29] that may be rather simple and have been found to be predictive for the response to song elements [28]. We cannot rule out the possibility that Field L2 already contributes to perceptual restoration. It is more likely, however, that the neural substrate for the observed behavioral response is found in secondary auditory areas of the starling forebrain, specifically the caudo-medial Mesopallium and the caudo-medial Nidopallium. In these areas of the starling forebrain that receive afferent input from the Field L complex [25], [27] neurons have been found that are able to act as complex non-linear feature detectors [19] that modify their responses when becoming familiar with a novel song [19]. These neurons could provide the basis for perceptual restoration and account for the different outcome of the familiar and unfamiliar condition in this study. Other candidate neurons for learning-dependent complex non-linear feature detectors that could contribute to the perceptual restoration of song elements can be found in the song system nucleus HVC [30], [31], [32], [33]. The neurons in this nucleus respond most strongly to bird's own song motifs with song notes in the natural order and less to motifs of con-specific songs. Since we found no difference how the starlings responded to familiar motifs that they had heard before and motifs that they produced themselves, the involvement of HVC in the observed perceptual restoration of familiar motifs is less likely. A study obtaining neural recordings from songbirds during the moment at which they may experience the illusionary percept will allow identifying the sites and mechanisms involved in the perceptual restoration of learned song motifs in birds that resemble the perceptual restoration of familiar speech signals in humans.

## Materials and Methods

### Subjects

The four adult male European starlings (*Sturnus vulgaris*) that were studied were caught in their natural environment near Munich, Germany, under a permit from the government of Upper Bavaria. Since the birds were taken from the wild, they had a normal experience with birdsong during their development. Prior to testing, the starlings were kept together in an outdoor aviary at Oldenburg University. During testing they were kept in individual cages (40×40×80 cm) in which they could hear each other. All starlings were naïve to psychophysical experiments. To motivate the starlings in the operant procedures their weight was kept at approximately 90% of their free-feeding weight. The care and the treatment of the animals were in accordance with the procedures of animal experimentation approved by the Government of Lower Saxony, Germany, through the Niedersächsisches Landesamt für Verbraucherschutz und Lebensmittelsicherheit (LAVES).

### Acquisition and manipulation of stimuli

For obtaining the motifs used in the familiar song condition, we recorded the songs of the starlings that participated in the experiments while they were in an outdoor aviary without any other starlings in the spring of 2006. Recording was done during the early morning hours (6–9 am, GMT). The maximum distance between bird and microphone was 2.5 m resulting in clear recordings with little background noise in the frequency range of the motifs. We used a solid-state recorder (sampling rate 44.1 kHz with a 16 bit resolution, Marantz PMD 670, Marantz, USA) and a directional microphone (ME 88, Sennheiser, Germany). The familiar motifs were either well known to the birds because a conspecific in the same aviary produced them or because they sung the motifs themselves (i.e. these motifs were from the bird's own song). The motifs used in the unfamiliar song condition were obtained by A/D conversion from starling songs recorded by M. Eens, Antwerp. These songs were also recorded from a short distance (often less than 1 m) at starling nest boxes in Belgium before 1989 and had little background noise. Considering the average lifetime of a European starling of 22 months [34] and the large distance between the two sites from which the birds originated and from which the recordings were obtained, it is highly unlikely that the tested Starlings were familiar with these Belgian songs.

To prepare the song motifs for testing, we high pass filtered (0.8 kHz cut off frequency) the songs and cut the variable part of the song [15] into single motifs (Avisoft SASLab pro, V4.36). We then normalized all motifs to the same root mean square level (58 dB SPL). These motives were the complete motifs. The ten motifs chosen from the familiar songs had a duration of 575 ms±17 ms (mean±SD), and the ten motifs from the unfamiliar songs had a duration of 555 ms±29 ms. For creating the gap motifs we inserted a silent gap with a duration of 50 ms every 75 ms (Fig. 1). For creating the noise motifs, a Hanning-ramped (5 ms) band-passed noise (0.8–22.5 kHz) with twice the RMS value of the motif replaced the silent gap. All modifications were done using custom MatLab software (Mathworks Inc, USA).

### Experimental setup

Experiments took place in a lighted sound-proof booth (IAC 401A, Industrial Acoustics Company, Germany) in which the walls were covered with additional sound absorbing material (Illbruck Illtec Pyramide 100/50, Illbruck Illtec PLANO Type 50/0). The reverberation time was only 12 ms (RT_60_) ensuring that the gaps in the motifs were not filled with reverberant sound. Stimuli were produced by a Linux computer via a D/A converter (16 bit, 44.1 kHz sampling rate, RME Multiface II, RME, Germany), attenuated to the appropriate level (58 dB SPL, PA5 programmable attenuator, TDT, USA), amplified (RMB 1048, Rotel, UK) and presented through a loudspeaker (KEF RDM one, KEF, UK, 100 Hz–18 kHz) positioned about 50 cm above the position of the bird's head in the experimental cage. The experimental cage (60×30×40 cm, mounted on a wire-frame shelf) had two perches - an upper perch on which the birds tended to sit spontaneously and a lower perch in front of a computer-operated feeder. Next to the feeder was a feeder light. The upper perch was equipped with a light barrier connected to the computer that allowed determining whether the bird sat on this perch.

### Experimental procedure

We trained the birds with an operant Go/NoGo procedure to indicate the detection of a deviator in the repeated background that was constant throughout an experimental session. For example, the background was one of the three motif modifications presented every 1.3 s while the deviators were the other two modifications. The bird started a trial by jumping onto the upper perch. It had to wait on the upper perch while the repeated background stimulus was presented. After a random waiting interval between 1 and 10 s a deviator replaced the background stimulus. The birds were trained to leave the upper perch to indicate the detection of the deviator. We measured the response latency of the birds to the deviator that was determined as the time period between the start of the deviator and the bird leaving the upper perch. If the response latency to the deviator was not more than 2 s, the feeder light was switched on for 5 s and the bird was rewarded with a piece of mealworm (beetle larva, *Tenebrio molitor*). In 40% of all trials only the feeder light (secondary reinforcer) was switched on for 5 s. If the bird left the upper perch without a deviant stimulus, the main light in the booth was switched off for 5 s and the trial was restarted. If the bird did not respond to a deviator a response latency of 2 s was scored and the next trial started without a reward. In the control trials, a background stimulus was played instead of a deviator and the response latency was measured as in trials with deviators.

### Baseline training

Initially, the birds learned to take food from the computer-controlled feeder. In the next step, they learned to discriminate pure tones (400 ms duration, frequency range 1100 Hz to 3492 Hz, logarithmically spaced in this range) with a highly salient difference in frequency. From one session to the next, the frequency of the background stimulus was changed to the frequency of a former deviator to support the birds' tendency for generalizing the discrimination task. To broaden the generalization, new stimulus sets such as sets composed of sinusoidally amplitude-modulated broadband noise (400 ms duration, modulation rate from 20 to 113 Hz, logarithmically spaced in this range) differing in modulation frequency or sets composed of starling frequency modulated whistles (600 ms duration, peak frequency range from 3.5 to 10.1 kHz) were used for the discrimination. If the birds responded correctly (i.e., left the perch after presentation of a deviator or stayed on the perch after presentation of a control) in at least four out of six trials per block of trials throughout at least eight out of ten blocks of the session, they were presented with the next background/deviator combination. This relatively complex criterion was chosen to encourage the birds to work continuously throughout the whole session. Given that criterion, the birds on average responded correctly in 82% of the trials. After finishing all possible background/deviator combinations of one stimulus set, the birds moved on to the next stimulus set. After going through an average of 36 sessions with the different stimulus sets they were presented with sets of motifs in the main experiment. These motifs had not been used in the baseline training.

### Main experiment

Each measurement for the perceptual distances between the modifications of a motif was based on one experiment consisting of three sessions with 90 trials each. In each of the three sessions, one of the modifications (complete, gap, noise) was presented as the background and the other two were the deviators. Deviators and controls were presented in a pseudo-random order in 30 blocks of 3 trials each in which the sequence of the motif modifications was randomized. Thus, combining the results from the three sessions 60 response times were obtained for discriminating between two different motif modifications and 30 response times for the control (spontaneous responding). The response time data from the discrimination were used for the multidimensional scaling analysis. To prevent the birds from responding to the noise pattern per se, we interspersed a noise pattern equal to that in the noise motif (i.e., only the noise of this stimulus without any motif parts) with a probability of 20% between the repeated background sounds and not overlapping with the motif modifications. Responding to the noise pattern alone resulted in a 5 s blackout as did any leaving of the upper perch during the presentation of the repeated background of motif modifications.

To ensure that the birds were sufficiently prone to respond to the deviators, we applied a criterion to every session for inclusion into the analysis derived as follows: after collecting data from one round of three sessions (i.e., one experiment) in which each had a different modification of the motif as the background, the overall number of all deviator detections per session was counted and the mean value of the performance of the four birds and the standard error were determined. If a bird responded to fewer deviators than this mean minus two times the standard error, the bird's response propensity was considered to be below average and this session was repeated once. This repeated session was then used for the subsequent analysis instead of the first session.

In the analysis of each experiment, we calculated the mean response time for every background/deviator combination. These data were fed into a multidimensional scaling procedure (PROXSCAL, SPSS 15, SPSS Inc.) to obtain a measure of the perceived similarity of the three modifications of the motif tested in the current experiment.
